# Does the mainland China–Hong Kong exchange program change the views of local university students in Hong Kong on regional cooperation? A randomized control-group pre-test post-test experiment

**DOI:** 10.3389/fpsyg.2023.1078437

**Published:** 2023-03-06

**Authors:** Jianzheng Liu, Jie Li, Xiwen Zhang, Jing Song, Weifeng Li, Jiansheng Wu

**Affiliations:** ^1^School of Public Affairs, Xiamen University, Xiamen, China; ^2^School of Tourism Management, Sun Yat-sen University, Zhuhai, China; ^3^School of Architecture and Urban Planning, Guangdong University of Technology, Guangzhou, China; ^4^School of Urban Planning and Design, Peking University Shenzhen Graduate School, Shenzhen, China; ^5^Department of Urban Planning and Design, Faculty of Architecture, The University of Hong Kong, Pokfulam, Hong Kong SAR, China; ^6^Shenzhen Institute of Research and Innovation, The University of Hong Kong, Shenzhen, China

**Keywords:** university students, Hong Kong, mainland China, exchange program, regional cooperation, randomized controlled experiment

## Abstract

The Chinese central government has been running an intensive exchange program called the Mainland China–Hong Kong Ten Thousand Student Exchange Program since 2012 to support local Hong Kong university students’ visits to mainland China, with the aim of promoting exchange and regional cooperation between Hong Kong and mainland China. However, little is known about local Hong Kong university students’ views on regional cooperation and whether the program is effective in changing their views. Using a randomized experimental design, we find that most students hold positive views on regional cooperation between Hong Kong and mainland China, but a considerable percentage of students oppose integration with mainland China. We also find that the program is effective in positively changing students’ views on certain aspects of regional cooperation related to the free trade zones and the Hong Kong–Zhuhai–Macau Bridge, but is not significantly effective on other aspects of regional cooperation. This study provides the first causal quantitative evidence regarding the impact of the mainland China–Hong Kong exchange program on local university students’ views regarding regional cooperation. The findings help inform the public about the prospect of regional cooperation and offer policy implications on youth exchange between mainland China and Hong Kong.

## 1. Introduction

The growing bias and hostility of young people in Hong Kong toward mainland China, as evidenced by the massive youth-led protests in Hong Kong in recent years (Cheng, 2016; Veg, 2017), have been endangering the existing connections and regional cooperation between Hong Kong and mainland China (Cheung, 2012; Ma, 2015; Chan, 2020; Lee and Chou, 2020). These negative sentiments and the resultant tensions, possibly driven by concerns over Hong Kong’s increasing political and economic dependence on mainland China (Ma, 2015) and the decline of political trust in the Chinese central government in the Hong Kong society (Steinhardt et al., 2018), have had detrimental impacts on regional cooperation between Hong Kong and mainland China, raising tremendous concerns in both regions. Mainland China is concerned about the implementation of many development initiatives that involve Hong Kong. The initiative of Guangdong–Hong Kong–Macao Greater Bay Area proposed by the Chinese government in early 2015, for example, aims to transform Hong Kong, Macao, and nine cities in Guangdong into a world-class city cluster for increasing the Chinese economy’s value adding capabilities and boosting internationalization (National Development and Reform Commission, 2015). However, the negative sentiments of Hong Kong youth and the resultant tensions may delay the implementation of this national strategic initiative in Hong Kong. Hong Kong is also worried about its own development because many of its leading industries rely heavily on regional cooperation with mainland China (Smart and Lin, 2004; Cheung, 2012; Shen and Luo, 2013). The negative sentiments and tensions may affect Hong Kong’s leading status in the greater Pearl River Delta region in the future and its competitive advantage over other cities in the region.

To address these concerns, the Chinese central government has been trying to attract Hong Kong young residents, especially local university students in Hong Kong, to visit mainland China with the aim of promoting exchange and cooperation between Hong Kong and mainland China (Fung and Leung, 2018; Xiang, 2018). In 2012, the Chinese central government launched a mainland China–Hong Kong exchange program called the Chinese Ministry of Education (MOE) Ten Thousand Student Exchange Program to support local Hong Kong university students’ visits to mainland China. To date, hundreds of thousands of exchange programs have been funded and implemented, with statistics from the MOE showing that over 10,000 local Hong Kong university students participate in the exchange programs each year (Ministry of Education, 2017). However, the effect of the program remains unknown. There is little research examining the views of local Hong Kong university students on regional cooperation between Hong Kong and mainland China or investigating the effect of the government-sponsored exchange program on these views.

This study seeks to address this research gap by examining local Hong Kong university students’ views on regional cooperation and ascertaining whether and to what extent the government-sponsored exchange program is effective in changing these views using a randomized control group pre-test post-test experimental design. The contribution of this study is that it provides the first causal evidence on the effects of the mainland China–Hong Kong exchange program on students’ views with regard to regional cooperation between Hong Kong and mainland China. The findings will help inform the public about the prospect of regional cooperation between Hong Kong and mainland China and offer policy implications on youth exchange between the two regions. In the next section, the study briefly introduces the mainland China–Hong Kong exchange program, followed by an introduction of the theoretical background and hypothesis. The experimental design is then described, including the participants, intervention, surveys, and data analysis methods. The subsequent section presents the results, followed by a discussion of the results and future research.

## 2. The mainland China–Hong Kong exchange program

The MOE Ten Thousand Student Exchange Program began at the centenary ceremony of The University of Hong Kong on 18 August 2011, when the then-Chinese-Vice-Premier Li Keqiang announced that the Chinese central government would set up a dedicated fund to support 1,000 students and teachers at the university to participate in study tours to mainland China every year (Xinhua News Agency, 2011). The program was successfully launched in 2012 and soon expanded to include seven other University Grant Committee (UGC)-funded universities in Hong Kong (The Chinese University of Hong Kong, The Hong Kong University of Science and Technology, Hong Kong Polytechnic University, City University of Hong Kong, Hong Kong Baptist University, Lingnan University, and The Education University of Hong Kong), The Hong Kong Academy for Performing Arts, and three self-funded post-secondary institutions (Chu Hai College of Higher Education, Hong Kong Shue Yan University, and The Open University of Hong Kong). The quota also expanded from 1,000 to more than 10,000 students and teachers *per annum*. In 2016, the program was further expanded to include five universities in Macau (University of Macau, Macao Polytechnic Institute, Macao Institute for Tourism Studies, Macau University of Science and Technology, and City University of Macau).

The mainland China–Hong Kong exchange program is a government-sponsored program that differs in several ways from the usual student exchange programs between universities and ordinary student trips from Hong Kong to mainland China. First, the program is officially funded and overseen by the Chinese government. Specifically, the Hong Kong, Macau, and Taiwan Affairs Office in MOE oversees the exchange program. While the student exchange programs between universities and trips to mainland China are usually organized and funded by the universities and students themselves, in the mainland China–Hong Kong exchange program, each student participant is supported by a subsidy of 550 RMB (roughly 80 USD) per day to cover their transport, accommodation, and meal expenses. Second, the mainland China–Hong Kong exchange program is specifically designed to target local university students in Hong Kong with the aim of promoting exchange and cooperation between mainland China and Hong Kong. MOE requires that the exchange should help local university students in Hong Kong to enhance their knowledge and understanding of mainland China, and promote exchanges and cooperation between mainland and Hong Kong (Xiang, 2018). Third, the mainland China–Hong Kong exchange program is usually organized in a rigorous and intensive way. Application for the exchange program opens twice each year. In each application window, two teachers from the applicant university in Hong Kong and its partner university in mainland China take charge of the application process and the study tour. Each application must contain a detailed daily plan of the study tour specifying the participants, hosts, time and place, purpose, and content of the activities, as well as the expected outcome. Lastly, students participating in the mainland China–Hong Kong exchange program are usually well-received and have an informative experience. The Chinese central government requires local government agencies, public institutions, enterprises, and non-profit organizations in mainland China to proactively receive local university students in Hong Kong who are participating in the exchange program. As an incentive measure, reception work related to Hong Kong is an important part of the performance review for public organizations in mainland China. Neither the student exchanges between universities nor ordinary trips to mainland China organized by students themselves usually afford the opportunity to engage with local government agencies, public institutions, enterprises, and non-profit organizations in mainland China.

The exchange program has proven to be very popular and are organized on a large scale. Statistics show that over 10,000 local Hong Kong university students participate in the exchange program and more than 170,000 visits from Hong Kong students are supported by similar programs each year (Ministry of Education, 2017).

## 3. Theoretical background and hypothesis

Two theoretical perspectives can explain the impacts of the exchange program on local university students in Hong Kong. The first perspective views the exchange program as contact between two groups with tensions. The intergroup contact theory proposes that the intergroup contact can reduce intergroup tensions and improve intergroup attitudes through increased positive emotions and decreased negative emotions (Allport, 1954; Tajfel and Turner, 1979; Miller et al., 2004; Hayward et al., 2017). Therefore, the intergroup contact theory can explain the impact of the exchange program on the attitudes of local Hong Kong university students toward mainland Chinese people. The second perspective views the exchange program as a social marketing program used to persuade a group of people to change their views on certain issues (Hovland et al., 1953; Greenwald, 1968; Petty and Cacioppo, 1986). Therefore, theories of persuasion can explain the role of exchange programs in changing the views of local Hong Kong university students on regional cooperation between mainland China and Hong Kong.

The most influential theory of persuasion is the message learning approach (also called the Yale approach) proposed by Hovland et al. (1953). The message learning approach proposes that persuasion for attitude change is most effective when the recipients attend to, comprehend and learn the message. Anything that helps facilitate the above processes would be beneficial for persuasion. For example, credible sources being provided to the recipients would increase persuasion through motivating recipients to learn the message (Petty and Cacioppo, 1986). But soon, extensive research revealed that learning of message was not the only mechanism of persuasion and there were other mechanisms such as recipients’ cognitive response to the message (Greenwald, 1968). The cognitive response theory proposed by Anthony Greenwald suggests that it is not the message itself that produces the persuasion and attitude change, but the cognitive response the recipients generated upon receiving the message (Greenwald, 1968). According to the cognitive response theory, recipients’ cognitive response can be influenced by the persuasion setting, source credibility and message content (Breckler and Wiggins, 1991). The cognitive response theory played a major role in subsequent development of persuasion theories. Much research found that variables such as source credibility or recipients’ moods have multiple effects on persuasion outcomes, and there were multiple mechanisms underlying these outcomes (Geers et al., 2018). To explain these diverse persuasion outcomes, Petty and Cacioppo (1986) built on the cognitive response theory and proposed the elaboration likelihood model. The elaboration likelihood model holds that persuasion works through multiple processes, some of the processes involve a lot of thoughts on the message (high-elaboration processes), whereas others involve little thought on the message (low-elaboration processes). Low-elaboration processes are referred as peripheral route to persuasion and high-elaboration processes are referred as central route to persuasion. The elaboration likelihood model suggests that message recipients’ ability and motivation to think determines whether they adopt the peripheral or the central route to persuasion. For example, when the perceived personal relevance of a message is high to the recipient, the recipient will have high motivation, and therefore pays more attention to examine the evidence in the message *via* the central route and will be persuaded to accept the message if the evidence is found to be strong (Petty and Cacioppo, 1979). Conversely, if the motivation to think is low, the recipient is usually less influenced by the quality of evidence in the message but more affected by simple cues in the message that takes less cognitive efforts to process, such as the attractiveness of the message source (Geers et al., 2018).

In the context of the mainland China–Hong Kong exchange program, the local Hong Kong University students may have comprehended and learned the message about enhancing regional cooperation between Hong Kong and mainland China during their conversations and discussions with urban planners, scholars and government officials in mainland China. Therefore, the empirical expectation of this study, which is formalized into the hypothesis, is that the mainland China–Hong Kong exchange program can positively change the views of local Hong Kong university students on regional cooperation between Hong Kong and mainland China.

## 4. Methods

### 4.1. Design and participants

**Figure 1:**
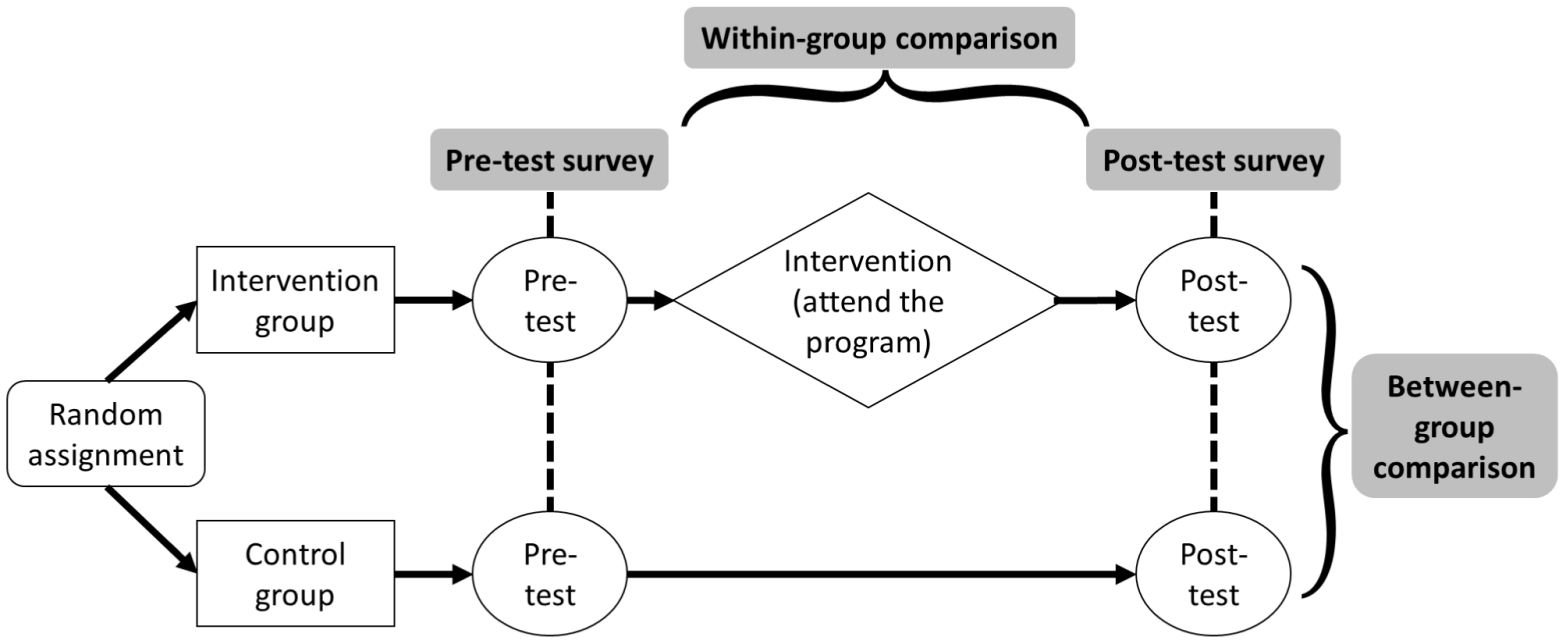
Randomized control group pre-test post-test experimental design.

This study used a randomized control group pre-test post-test design (Figure 1), which is a classic controlled experimental design with both a control group and an intervention group. This design is strong in establishing causal relationships because it randomly assigns participants to an intervention group or control group (Shadish et al., 2002). Using this design, data and measurements were collected through surveys at pre-test (baseline) and post-test (8 weeks follow-up).

The study was conducted on the campuses of The University of Hong Kong. The criterion for participant recruitment was local full-time undergraduate students of Chinese ethnicity. For practical and ethical reasons, neither random sampling nor systematic sampling was feasible in this study, therefore voluntary sampling was used to recruit participants. We first put up posters on all campuses of The University of Hong Kong to invite students to register. After the registration was completed, we then excluded ineligible students and used simple randomization to assign students to the intervention group or control group.

Three hundred and thirty six students registered to attend the exchange program. After excluding students who were either postgraduate students, part-time students, or non-local students, 44 and 37 students were enrolled in the intervention and control groups, respectively. As the calculation of the required sample size depends on the outcome variable of this study (students’ views on regional cooperation) (Dong and Maynard, 2013; Gruener, 2020), and the outcome variable has not been quantified before, we have to rely on expert estimates to determine the required sample size. It is suggested that a sample of 10–20 can yield relatively high statistical power to detect moderate differences between groups for experimental research in the literature (Roscoe, 1975; Sekaran and Bougie, 2016). Thus, the current sample size is considered appropriate for this pilot study.

### 4.2. Intervention

The students in the intervention group received the intervention of attending the exchange program, while the students in the control group remained in Hong Kong. Each student in the intervention group was required to carry a cell phone with a Hong Kong mobile phone number. They were also allowed to carry laptops and other personal electronic devices. This enabled the students in the intervention group to exchange messages and surf the Internet freely even when they were in mainland China as mobile phone service providers and The University of Hong Kong have been providing virtual private network services that could help students bypass the Great Firewall of China. All expenses, including transportation, accommodation, and meals, were covered by the exchange program and the students did not pay any expenses related to the study tour. The tour destinations comprised cities in the Pearl River Delta, including Shenzhen, Guangzhou, Zhaoqing, and Zhuhai. During the exchange, students attended tours to urban villages, industrial zones, and free trade zones, and round table discussions with government officials, policy analysts, urban planners and researchers in mainland China on regional planning, transportation, and environmental protection. Supplementary Table S1 shows a detailed description of the exchange program.

### 4.3. Data collection and measures

The questionnaire collected a wide variety of information from the students, including demographic data; their interest in and knowledge of cities and urban development in mainland China; and their views on regional cooperation between Hong Kong and mainland China. In this paper, we focus on students’ views on regional cooperation between Hong Kong and mainland China. Specifically, we asked six questions (Table 1). All question items used a four-point Likert scale ranging from strongly disagree to strongly agree. For convenience, this study used a unique ID to denote each question. For example, A1 refers to the item “The relationships and connections between Hong Kong and mainland China on regional planning are important.”

**Table 1:** Question items for measuring the students’ views on regional cooperation between Hong Kong and mainland China.

Variable ID	Questions/Statements	Response
A1	The relationships and connections between Hong Kong and mainland China on regional planning are important.	4-point Likert scale from strongly disagree to strongly agree
A2	There is more cooperation than competition between Hong Kong and mainland China regarding the economy.	Same as above
A3	The Hong Kong–Zhuhai–Macau Bridge offers more advantages than disadvantages to Hong Kong	Same as above
A4	Hong Kong should strengthen cooperation with mainland China in terms of regional environmental protection, regional transportation and regional logistics.	Same as above
A5	The initiation of Free Trade Zones in Guangdong, such as Qianhai in Shenzhen, Nansha in Guangzhou and Hengqin in Zhuhai, helps extend cooperation between Hong Kong and mainland China.	Same as above
A6	Hong Kong and Macau should be included in the Pearl River Delta regional planning in mainland China.	Same as above

Questionnaire surveys were administered to all students at the main campus of The University of Hong Kong using a computer-assisted approach at the pre-test stage 1 week before the intervention and at the post-test stage 7 weeks after the intervention. The survey was approved by the Human Research Ethics Committee for Non-Clinical Faculties at The University of Hong Kong. Each participant read and signed the informed consent form before participating in the survey. Each participant was rewarded with a 60 HKD (approximately 8 USD) cash coupon.

To better understand the effect of the exchange program, we devised two binary outcome variables for each question item: increased agreement and changed views. Increased agreement was present if a participant reported a higher-ordered Likert value at the post-test stage than at the pre-test stage. For instance, if a participant reported ‘agree’ at the pre-test stage and ‘strongly agree’ at the post-test stage for a question item, then increased agreement was present for that question item and the variable was coded as 1. A changed view was present if an individual changed his or her view from disagree to agree. For instance, if a participant reported ‘disagree’ at the pre-test stage and ‘agree’ at the post-test stage for a question item, then a changed view on the question item was present and the variable was coded as 1.

### 4.4. Data analysis

Results are presented as frequencies and percentages for categorical data. For continuous data, means with standard deviations are presented. Chi-square tests or Fisher’s exact tests were used to compare baseline characteristics on categorical variables between the intervention group and control group, while differences in continuous variables were analyzed using two-sample *t-*tests. Wilcoxon–Mann–Whitney tests were used to assess the differences in students’ views on regional cooperation between the two groups at the pre-test stage (baseline). For the differences between the pre-test and post-test within the same group, we used the Wilcoxon signed rank sum test.

To assess the effectiveness of the exchange program, odds ratios with 95% confidence intervals (CIs) for the two binary outcome variables (i.e., increased agreement and changed views) were calculated for each question item. For rare outcomes, the confidence intervals for the odds ratios were calculated using the mid-p method (Fagerland, 2012). All statistical analyses were conducted in Stata 14^1^. Results were considered statistically significant at a *p-*value of less than 0.05.

## 5. Results

### 5.1. Students’ views on regional cooperation between Hong Kong and mainland China

A comparison of the baseline demographic characteristics between the intervention group and control group is shown in Table 2. The baseline demographic characteristics between the two groups did not differ significantly for all demographic variables.

**Table 2:** Demographic characteristics of students in the intervention group and control group.

Participant’s demographic characteristics	Intervention group (*n* = 44)	Control group (*n* = 37)	*p*-values for the testing of differences between the two groups
Age, mean (SD), year	19.9 (1.5)	20.1 (1.6)	0.676
Gender			0.296
Male, *n* (%)	18 (40.9)	11 (29.7)	
Female, *n* (%)	26 (59.1)	26 (70.3)	
Household income			0.229
Under 10,000 HKD, *n* (%)	3 (6.8)	8 (21.6)	
10,000–30,000 HKD, *n* (%)	21 (47.7)	17 (45.9)	
30,000–50,000 HKD, *n* (%)	14 (31.8)	7 (18.9)	
Above 50,000 HKD, *n* (%)	6 (13.6)	5 (13.5)	
Subject area			0.507
Social science and humanities, *n* (%)	24 (54.5)	17 (45.9)	
Natural science, *n* (%)	20 (45.5)	20 (54.1)	

Means with the standard deviations (SDs) are presented for the continuous variable, and numbers with the percentages of students are presented for categorical variables. The *p*-values for continuous variables, such as age, are analyzed using two-sample *t-*test, and the *p*-values for categorical variables are analyzed with the Chi square test or Fisher’s exact test. A value of *p* < 0.05 denotes statistical significance.

Table 3 shows the baseline characteristics of the students’ views on regional cooperation between Hong Kong and mainland China (see the columns labelled ‘Pre-test n (%)’ in Table 3). The results show that most of the students held positive views on regional cooperation between Hong Kong and mainland China. An overwhelming majority of students agreed or strongly agreed that the connections and cooperation between Hong Kong and mainland China were important for Hong Kong. Most students agreed that more economic cooperation than competition occurred between Hong Kong and mainland China, and the Hong Kong–Zhuhai–Macau Bridge offered more advantages than disadvantages to Hong Kong. Most students believed that the free trade zones in Guangdong would help extend cooperation between Hong Kong and mainland China, and that both parties should strengthen cooperation. However, a considerable percentage of students (31.9% in the intervention group and 35.1% in the control group) disagreed that Hong Kong and Macau should be included in the Pearl River Delta regional planning in mainland China.

**Table 3:** Students’ views on regional cooperation between Hong Kong and mainland China before the intervention (pre-test) and after the intervention (post-test) among the intervention group and control group.

Variables	Students’ views	Intervention group (*n* = 44)			Control group (n = 37)		
		Pre-test *n* (%)	Post-test *n* (%)	*p*	Pre-test *n* (%)	Post-test *n* (%)	*p*
A1	Strongly disagree	0 (0)	0 (0)	0.371	0 (0)	0 (0)	0.739
	Disagree	2 (4.5)	5 (11.4)		4 (10.8)	1 (2.7)	
	Agree	37 (84.1)	27 (61.4)		27 (73)	32 (86.5)	
	Strongly agree	5 (11.4)	12 (27.3)		6 (16.2)	4 (10.8)	
A2	Strongly disagree	1 (2.3)	0 (0)	0.796	0 (0)	0 (0)	0.234
	Disagree	8 (18.2)	7 (15.9)		4 (10.8)	3 (8.1)	
	Agree	29 (65.9)	33 (75)		26 (70.3)	33 (89.2)	
	Strongly agree	6 (13.6)	4 (9.1)		7 (18.9)	1 (2.7)	
A3	Strongly disagree	1 (2.3)	0 (0)	0.361	1 (2.7)	1 (2.7)	0.706
	Disagree	9 (20.5)	4 (9.1)		9 (24.3)	10 (27)	
	Agree	26 (59.1)	35 (79.5)		24 (64.9)	23 (62.2)	
	Strongly agree	8 (18.2)	5 (11.4)		3 (8.1)	3 (8.1)	
A4	Strongly disagree	0 (0)	0 (0)	0.251	0 (0)	0 (0)	0.031
	Disagree	2 (4.5)	1 (2.3)		0 (0)	2 (5.4)	
	Agree	22 (50)	29 (65.9)		20 (54.1)	25 (67.6)	
	Strongly agree	20 (45.5)	14 (31.8)		17 (45.9)	10 (27)	
A5	Strongly disagree	1 (2.3)	0 (0)	<0.001	0 (0)	0 (0)	0.706
	Disagree	13 (29.5)	1 (2.3)		2 (5.4)	2 (5.4)	
	Agree	30 (68.2)	35 (79.5)		33 (89.2)	32 (86.5)	
	Strongly agree	0 (0)	8 (18.2)		2 (5.4)	3 (8.1)	
A6	Strongly disagree	5 (11.4)	2 (4.5)	0.211	1 (2.7)	0 (0)	0.617
	Disagree	9 (20.5)	10 (22.7)		12 (32.4)	9 (24.3)	
	Agree	23 (52.3)	24 (54.5)		20 (54.1)	27 (73)	
	Strongly agree	7 (15.9)	8 (18.2)		4 (10.8)	1 (2.7)	

Numbers with the percentages of students are presented for each ordinal variable in the pre-test and post-test. A value of *p* < 0.05 denotes statistical significance. The columns labelled ‘*p*’ denote the *p* values for the testing of within-group differences between the pre-test stage and the post-test stage using Wilcoxon signed rank sum test.

### 5.2. The impacts of the exchange program on students’ views

Table 3 also shows the within-group differences between the pre-test stage and the post-test stage for both groups. After attending the exchange program, significantly more students in the intervention group agreed or strongly agreed that the free trade zones in Guangdong helped extend cooperation between Hong Kong and mainland China. More students in the intervention group agreed or strongly agreed that there was more cooperation than competition between Hong Kong and mainland China regarding the economy, the Hong Kong–Zhuhai–Macau Bridge offered more advantages than disadvantages to Hong Kong, Hong Kong should strengthen cooperation with mainland China, and that Hong Kong and Macau should be included in the Pearl River Delta regional planning in mainland China, but the differences between the pre-test stage and the post-test stage were not significant.

Although these within-group comparisons might suggest certain insights, the within-group differences were not sufficient to justify that the changes had been caused by the program because these differences are subject to various threats to internal validity (Knapp, 2016). Only the differences between the changes in the intervention group and the changes in the control group can be attributed solely to the influence of the program’s effect. Table 4 shows the statistical analysis results of the two binary outcome variables (increased agreements and changed views from disagree to agree) on regional cooperation between Hong Kong and mainland China in the intervention group and control group. The odds ratios with 95% confidence intervals for each question item are presented in Table 4.

**Table 4:** Increased agreement and changed views from disagree to agree of students on regional cooperation in the intervention and control groups.

Variable		Intervention (*n*, %)		Control (*n*, %)		*p*	Odds ratio (95% CI)	
A1	Increased agreement	12	(27.3)	5	(13.5)	0.13	2.4	(0.78–7.29)
	Changed views	2	(4.5)	4	(10.8)	0.283	0.39	(0.00–1.97)
A2	Increased agreement	8	(18.2)	4	(10.8)	0.352	1.83	(0.53–6.27)
	Changed views	4	(9.1)	4	(10.8)	0.769	0.83	(0.21–3.26)
A3	Increased agreement	11	(25.0)	3	(8.1)	0.045	3.78	(1.03–13.66)
	Changed views	9	(20.5)	2	(5.4)	0.049	4.5	(1.01–21.62)
A4	Increased agreement	7	(15.9)	3	(8.1)	0.288	2.14	(0.55–8.21)
	Changed views	1	(2.3)	0	(0)	0.356	2.59	Not available
A5	Increased agreement	18	(40.9)	4	(10.8)	0.002	5.71	(1.79–18.02)
	Changed views	14	(31.8)	2	(5.4)	0.003	8.17	(1.80–37.68)
A6	Increased agreement	11	(25.0)	9	(24.3)	0.944	1.04	(0.38–2.80)
	Changed views	5	(11.4)	8	(21.6)	0.21	0.46	(0.14–1.51)

Table 4 shows that, compared with the control group, the intervention group had higher proportions of increased agreement for all question items. However, significantly higher percentages of increased agreement in the intervention group than in the control group only occurred for A3 (*p* = 0.045) and A5 (*p* = 0.002). The odds ratios for A3 and A5 were 3.78 and 5.71, respectively. The results mean that compared with students who did not attend the exchange program, significantly higher percentages of students who participated in the exchange program had increased agreement on that the Hong Kong–Zhuhai–Macau Bridge offers more advantages than disadvantages to Hong Kong and that the free trade zones in Guangdong help extend cooperation between Hong Kong and mainland China. The odds that the students who attended the exchange program would present increased agreement on that the free trade zones in Guangdong help extend cooperation between Hong Kong and mainland China was 4.71-times greater than that of students who did not attend the exchange program. In response to the statement that the Hong Kong–Zhuhai–Macau Bridge offers more advantages than disadvantages to Hong Kong, the odds of having increased agreement was 2.78-times greater for students who had attended the exchange program than for those who had not. It is important to note that this study conducted multiple statistical tests to find possible significant results, which involves multiple testing problem. When a Bonferroni Correction is applied to adjust the alpha level, then significantly higher percentages of increased agreement only occurred for A5. However, one of the goals of this pilot experimental study is to explore possible significant results for further investigation. Therefore, this study did not correct for multiple testing.

For the outcome variable of changed views, the odds of changing from disagree to agree for the statement that the free trade zones in Guangdong help extend cooperation between Hong Kong and mainland China was 7.17-times greater for students who had attended the exchange program than for those who had not. The odds of changing from disagree to agree for the statement that the Hong Kong–Zhuhai–Macau Bridge offers more advantages than disadvantages to Hong Kong was 3.5-times greater for students who had attended the exchange program than for those who had not.

## 6. Discussion

### 6.1. Interpretation and implications

This study finds that the majority of student participants hold positive views on regional cooperation between Hong Kong and mainland China, but a considerable percentage of students disagree that Hong Kong and Macau should be included in the Pearl River Delta regional planning in mainland China. The results suggest that many local university students in Hong Kong support regional cooperation between Hong Kong and mainland China, but oppose regional integration with mainland China. On the one hand, local university students in Hong Kong know that the connections and cooperation between Hong Kong and mainland China are important for Hong Kong. Therefore, the students believe that Hong Kong should strengthen its cooperation with mainland China. On the other hand, local Hong Kong university students do not prefer Hong Kong to be assimilated into mainland China (Ye, 2021). They believe that Hong Kong’s advantage depends on its institutions, and Hong Kong should maintain its unique position and avoid losing these advantages to cities in mainland China (Cheung, 2012; Yang and Li, 2013). Therefore, they oppose regional integration with mainland China.

This study also finds that the exchange program is effective in changing students’ views on certain aspects of regional cooperation between Hong Kong and mainland China, such as the Hong Kong–Zhuhai–Macau Bridge offering more advantages than disadvantages to Hong Kong and the free trade zones extending cooperation. The reason behind is probably that the free trade zones and the Hong Kong–Zhuhai–Macau Bridge as well as their impacts were more visible to the students, and the students received the relevant information very well during the exchange program, thus making the program significantly effective in changing students’ views on the free trade zones and the Hong Kong–Zhuhai–Macau Bridge.

For other aspects of regional cooperation such as Hong Kong and Macau being included in the Pearl River Delta regional planning in mainland China, higher percentages of students who attended the exchange program increased their levels of agreement than those who did not, but the differences are not statistically significant. According to theories of persuasion, many aspects of the message source (e.g., credibility) and the recipient (e.g., social network) have impacts on persuasion outcomes (O'keefe, 2015; Geers et al., 2018). Two major factors related to the message source and the recipients probably contributed together to the failure in changing students’ views on these aspects of regional cooperation during the exchange program.

First, the perceived credibility of the message source (i.e., the people and institutions in mainland China who received the Hong Kong students during the exchange in this study) is not high for Hong Kong students, which does not help increase students’ acceptance of the message on regional cooperation. Although most Hong Kong students accept their Chinese ethnic and cultural identity, mainland China did not gain much of the trust and acceptance of many Hong Kong students, and many Hong Kong students probably viewed the messages from mainland China negatively as part of the propaganda (Ho and Tang, 2020). Therefore, the students were probably aware that they might be given censored information during the exchange program and were skeptical about the source of the message. As Professor Stephen W. K. Chiu put it (Chiu et al., 2017), “no matter whether what the Hong Kong young people see and hear during their visit to mainland China is true or not, they may look at mainland China through ‘tinted glasses’ with biases and prejudices.”

Second, students are likely to be influenced by their local social network in Hong Kong, which may disapprove certain aspects of regional cooperation between Hong Kong and mainland China. Studies have shown that many Hong Kong students are deeply involved in various social networks in Hong Kong, including schools’ alumni networks (Yuen and Tang, 2021) and online communities and networks through social media platforms (Chan and Fu, 2017; Chan et al., 2021). These networks were extensively used to mobilize students and teenagers during protests, demonstrations and riots in Hong Kong in recent years (Erni and Zhang, 2022). According to the elaboration likelihood model, when recipients do not invest a lot of thoughts on the message, they take the peripheral route to persuasion (low-elaboration processes) and their reactions to the message will be guided by simple heuristic principles such as the reactions of other people around them (“if other people do not believe it, then it’s probably false”) (O'keefe, 2015). Therefore, the student participants in this study probably did not think deeply about the regional cooperation between Hong Kong and mainland China. They just simply followed the negative opinions and reactions of their local social networks towards mainland China, and thus disapproved certain aspects of regional cooperation.

In addition, the research findings of this study could help inform policy making on regional cooperation in similar situations around the world, especially cross-border cooperation and integration in border regions with potential conflicts. Some of the potential regions where cross-border cooperation and integration play an important role in shaping the development and prosperity of the region include the China-Korea-Japan border regions in Northeast Asia (Yoshimatsu, 2010), the US–Mexico border regions in North America (Hanson, 2001), Europe (Brenner, 1999), and Southeast Asia (Bunnell et al., 2006). This study suggests that for these regions, a government-sponsored social intervention program may help promote cross-border cooperation.

### 6.2. Generalizability

As the exchange program requires student participants to self-select and join in, this study had to use voluntary sampling to recruit local university students in view of practical and ethical consideration. As a result, the external validity (generalizability) of the research findings of this study is limited. Strictly speaking, the findings of this study are applicable to local undergraduate students at The University of Hong Kong who are willing to participate in the exchange program. However, we would like to point out that many experimental studies of social intervention programs have the same issue of limited external validity because the social intervention programs under investigation require participants to self-select and join in due to ethical and practical reasons (Brady and O'Regan, 2009; Susiloretni et al., 2013; Andersen and Moynihan, 2018). These experimental studies of social intervention programs operate with this limitation and still have made important contributions.

We explored the generalizability of the research findings to other student populations by comparing the study population with the undergraduate student population in all eight UGC-funded universities in Hong Kong. We collected the statistical information provided by the University Grants Committee of the Hong Kong Special Administrative Region (The University Grants Committee, 2016; The University Grants Committee, 2019), and conducted comparisons and tests of differences between these groups in terms of demographic characteristics. As Supplementary Table S2 shows, there are no statistically significant differences in terms of demographic characteristics (age, gender, and subject) between the undergraduate students who participated in this study, the undergraduate student population at The University of Hong Kong, and the undergraduate student population at eight UGC-funded universities in Hong Kong, except that there are marginally significant differences in terms of gender between the students who participated in this study and the student population at The University of Hong Kong. Therefore, we can reasonably infer that the research findings of this study may also be applicable to local undergraduate students in all eight UGC-funded universities in Hong Kong who are willing to participate in the exchange program. An important caveat is that due to data unavailability, we did not compare the study sample with the undergraduate student population in all eight UGC-funded universities in Hong Kong in terms of household income and other important socioeconomic variables. Therefore, it is unknown whether there are significant differences between the student groups in terms of household income and other important socioeconomic variables, and whether the effect of the exchange program on students varies significantly depending on students’ socioeconomic background. Thus, the application of the findings in this study to other student populations in Hong Kong should be done cautiously.

### 6.3. Future research

This study has several limitations which point to avenues for future research. First, this study might be underpowered due to the limited sample size. Therefore, small effects might not have been detected, and a replication study with a larger sample size is needed. Moreover, a larger sample with stratified randomization would allow us to conduct a credible subgroup analysis to find out if the exchange program was more effective for some students than others. Second, similar to many experimental studies of social intervention programs that require actions by the participants and involvement of research staff (Brady and O'Regan, 2009; Susiloretni et al., 2013; Andersen and Moynihan, 2018), the random assignment in this experiment was neither blind to the experimenters nor blind to the participants due to practical and ethical reasons. To control for the potential influence of experimenters’ expectation, two local student assistants were hired in this study to avoid experimenter-participant contact as possible as we can. However, response bias (i.e., the tendency for participants in the intervention group to provide responses in a way they think would satisfy the experimenters) may exist due to a lack of blinding in this experiment. Third, history might account for the effect of the intervention in a typical randomized control group pre-test post-test design because events occurring concurrently with the intervention might have caused the observed effect. However, we examined the major newspapers in Hong Kong and mainland China during the intervention period and did not find any particular major events that might have affected students’ views. In addition, the findings of this study are based on the data collected in 2015. Students’ views on regional cooperation between Hong Kong and mainland China might have changed in other years. Therefore, generalization of the research findings to other years should be done with caution. The limitations above thus leave much room for future research.

## Data availability statement

The original contributions presented in the study are included in the article/Supplementary material, further inquiries can be directed to the corresponding author.

## Ethics statement

The studies involving human participants were reviewed and approved by the Human Research Ethics Committee for Non-Clinical Faculties at The University of Hong Kong. Written informed consent to participate in this study was provided by the participants’ legal guardian/next of kin. Written informed consent was obtained from the individual(s), and minor(s)’ legal guardian/next of kin, for the publication of any potentially identifiable data included in this article.

## Author contributions

JZL conceived the project, collected and analyzed the data, and wrote the manuscript. JL collected the data and contributed to the theoretical development. WL supervised the project and reviewed and edited the manuscript. XZ, JS, and JW collected the data and participated the data analysis. All authors contributed to the article and approved the submitted version.

## Funding

This work was supported by the National Natural Science Foundation of China (Grant No. 42101199), the Social Science Research Base Program of Fujian at the Research Centre of Public Service Quality of Xiamen University (Grant No. FJ2020JDZ006), the Fundamental Research Funds for the Central Universities (20720201032) in China, Guangdong Basic and Applied Basic Research Foundation (2019A1515111145) and The University of Hong Kong Seed Fund for Basic Research (201611159296).

## Conflict of interest

The authors declare that the research was conducted in the absence of any commercial or financial relationships that could be construed as a potential conflict of interest.

## Publisher’s note

All claims expressed in this article are solely those of the authors and do not necessarily represent those of their affiliated organizations, or those of the publisher, the editors and the reviewers. Any product that may be evaluated in this article, or claim that may be made by its manufacturer, is not guaranteed or endorsed by the publisher.

## Footnotes

^1^ https://www.stata.com
