# Supplementary material for: Does the mainland China–Hong Kong exchange program change the views of local university students in Hong Kong on regional cooperation? A randomized control-group pre-test post-test experiment
Source: Front Psychol. 2023 Mar 6;14:1078437. doi: 10.3389/fpsyg.2023.1078437 (PMC10025535; doi:10.3389/fpsyg.2023.1078437)
Supplement: Supplementary file 1 [file Data_Sheet_1.docx]

***Supplementary Material* *for***

Does the mainland China–Hong Kong exchange program change the views of local university students in Hong Kong on regional cooperation? A randomized control-group pre-test post-test experiment

This file includes Tables S1 to S2.

**Table S1. Details of the study tour**

| **Time** | **Place** | **Host** | **Activities** |
| --- | --- | --- | --- |
| Morning of Day 1 | Shenzhen | Shenzhen Urban Planning Exhibition Hall | Students attended a guided exhibition of the history of the urban development of Shenzhen and its plans for CBDs and new towns. Students conversed with the staff during the exhibition. |
| Late morning of Day 1 | Shenzhen | School of Urban Planning and Design of Peking University | Students attended a lecture titled ‘Planning for the Coordinated Development of the Greater Pearl River Delta Township’ and interacted with the speaker during the Q&A session. |
| Afternoon of Day 1 | Shenzhen | The Authority of Qianhai free trade zone, Shenzhen | Students attended a guided exhibition of the development and construction, operations management, investment promotion, and institutional innovation of the Qianhai free trade zone, then attended a tour to the construction field and conversed with staff of the Authority. |
| Morning of Day 2 | Guangzhou | Guangzhou Institute of Environmental Protection | Students attended a roundtable discussion with the executive director, policy analysts, and program managers of the institute and discussed the policies and measures on air quality and water quality in Guangzhou, relevant regional cooperative measures, as well as their implications for Hong Kong’s water supply and air quality. |
| Afternoon of Day 2 | Guangzhou | School of Geography and Planning of Sun Yat-sen University | Students attended two tours guided by a researcher from the host to two well-known urban villages in Guangzhou - Shipai village, which is awaiting renovation, and the renovated Liede village, and learned about the history and renovation development of urban villages, as well as residents’ lives in urban villages. |
| Late afternoon of Day 2 | Guangzhou | Xiaozhou village | Students attended a tour to the Xiaozhou village (a provincial model ecological village) and learned about local ecotourism and natural and architectural conservation. |
| Morning of Day 3 | Zhaoqing | Zhaoqing High-Tech Industry Development Zone | Students attended a tour to the Zhaoqing High-Tech Industry Development Zone and conversed with staff on the region’s strategies and plans on industrial upgrading, high-tech industry development, land use and transportation. |
| Afternoon of Day 3 | Zhaoqing | Yuejiang Tower, Chongxi Tower, Memorial Temple of Lord Bao | Students attended three tours to Yuejiang Tower, Chongxi Tower, and Memorial Temple of Lord Bao and learned about local cultural heritage and protection. |
| Morning of Day 4 | Zhuhai | The Authority of Hengqin free trade zone, Zhuhai | Students attended a guided exhibition of the spatial layout, industrial development plan, and ecological development plan of the Hengqin free trade zone as well as its strategy to strengthen the cooperation between Guangdong, Hong Kong, and Macau. |
| Afternoon of Day 4 | Zhuhai | Zhuhai Institute of Urban Planning and Design | Students attended a roundtable discussion with planners in the Zhuhai Institute of Urban Planning and Design and discussed regional transportation planning and its implications for cities in the Pearl River Delta. |

**Table S2. Testing of the differences in demographic characteristics (age, gender, and subject area) between student participants in this study, all full-time undergraduate students at The University of Hong Kong in 2015, and all full-time undergraduate students at eight universities funded by University Grants Committee in Hong Kong in 2015**

| **Participant’s demographic characteristics** | **Group 1-Students who participated in this study**  **(n=81)** | | **Group 2- All full-time undergraduate students at The University of Hong Kong**  **(n=15411)** | | **Group 3- All full-time undergraduate students at University Grants Committee-funded universities in Hong Kong (n=79369)** | | **p values for the testing of differences between Group 1 and Group 2** | **p values for the testing of differences between Group 1 and Group 3** |
| --- | --- | --- | --- | --- | --- | --- | --- | --- |
| Age, mean (SD), year | 20.0 | (1.5) | Not Available | | 20.2 | (1.7) | Not available | 0.247 |
| Gender |  |  |  |  |  | | **0.045** | 0.074 |
| Male, n (%) | 29 | (35.8) | 7252 | (47.1) | 36549 | (46.1) |  |  |
| Female, n (%) | 52 | (64.2) | 8159 | (52.9) | 42820 | (53.9) |  |  |
| Subject area |  |  |  |  |  | | 0.824 | 0.371 |
| Social Science, Art, and Humanities, n (%) | 41 | (50.6) | 7560 | (49.0) | 44313 | (56.0) |  |  |
| Natural Science, n (%) | 40 | (49.4) | 7851 | (51.0) | 35055 | (44.0) |  |  |

Means with the standard deviation (SD) are presented for the continuous variable, and numbers with the percentage of participants are presented for categorical variables. The p-values for age are analyzed using two independent samples t tests, and the p-values for categorical variables are analyzed with the Chi square test or Fisher’s exact test. A value of p<0.05 denotes statistical significance.
